# The Hospital. Nursing Section

**Published:** 1904-06-11

**Authors:** 


### The Hospital.

### Nursing Section.

Contributions for this Section of "The Hospital" should be addressed to the Editor, "The Hospital" Nursing Section, 28 & 29 Southampton Street, Strand, London, W.C.

No. 924.—Vol. XXXVI. *SATURDAY, JUNE* 11, 1904.

### Notes on News from the Nursing World.

### NURSING QUESTIONS IN THE HOUSE OF COMMONS.

On Monday Mr. Tennant elicited from the First Lord of the Treasury an intimation that he hoped a Select Committee on Nursing and the State Registration of Nurses might be appointed shortly, and he suggested that the two Bills before the House might be formally read a second time for the purpose of referring them to such a committee. The Government, he added, would not put any obstacle in their way, but he could not promise Government time. On the same day Sir J. Colomb asked the Secretary for War whether any communications have been issued from the War Office containing proposals to provide military stations with a nurse trained in maternity and general nursing to attend the wives and families of soldiers without expense to them, and, if so, to whom were such communications made, and what was their general purport. Mr. Arnold-Forster, answering the question, stated that letters were sent to all general officers commanding asking for an expression of opinion as to the best means of providing nursing for the wives and families of soldiers in places where hospitals were not provided. The replies, he added, have not yet been received from all the officers concerned.

### STATE REGISTRATION AT THE POINT OF THE SWORD.

An extraordinary attempt was made, at the meeting of the Royal British Nurses' Association on Monday afternoon, to force State registration forward at the point of the sword. Dr. Bedford Fenwick seconded a motion calling upon honorary officers of the Association who had signed the manifesto against registration to resign their membership of the Association. We are glad to say that this piece of intolerance was resented as it deserved to be. Only two votes were cast in favour of the motion, which was deprecated by the Chairman. If Dr. Bedford Fenwick had wished to do the cause he advocates a bad turn, he could not have attempted a more adroit method of discrediting it.

### ENGLISH NURSES AT THE BERLIN CONGRESS.

The list of papers which are to be read at the meeting of nurses in connection with the International Congress of Women at Berlin next week is remarkable for lack of special interest to English nurses. The name of one English ex-matron only appears on it, and her subject, "Trained Nursing as a Profession for Women, from an Educational, Economic, and Social Aspect," is so big hat no paper of reasonable length can do it justice. The United States nurses are much to the fore. Miss L. L. Dock, instead of roaming from China to Peru, finds "Trained Nursing in America" a sufficiently large topic; Mrs. Goodrich is to deal with a kindred topic; Miss Maud Banfield proposes to lay stress on the old maintenance of American nurses; and Miss Mary Thornton will take a prominent part in the subsequent discussion. Papers on "The Future Improvement of German Nurses," and "The Organisation of Trained Nurses in Sweden," will be read respectively by Sister Agnes Karll of Berlin, and Dr. Ellen Sandelin of Sweden.

### NEW HOME FOR DISTRICT NURSES AT LIVERPOOL.

On Friday last a new home for the accommodation of nurses belonging to the Liverpool Queen Victoria District Nursing Association was opened by the Lady Mayoress. The ceremony was preceded by a speech from Mr. Archibald Williamson, chairman of the Association, who gave a brief history and description of the district nursing movement since it was founded in 1862 by the late Mr. William Rathbone, mentioned the gifts of a number of generous subscribers, and announced that the home was opened free of debt. Mr. Herbert Rathbone was among the speakers. The home, which has a large garden attached, is situated in an open district, and is admirably arranged. The floors and the stair carpets are moss green, the woodwork being of ivory white to match the walls, on which is a pattern of green leaves. The bedrooms are complete, with appropriate furniture. The sitting-rooms have yellow walls and deep window seats, and the dining-room is in tones of deep red and brown. The district-room is fitted with cupboards, where each nurse keeps all the necessaries for her work and her district. There are also ample conveniences for disinfecting clothes in case of need.

### THE LESSON OF THE SPONGE CASE.

There is no doubt that Miss May Thorne, a lady surgeon, has been cast in damages, £25 and heavy costs, owing to the failure of the nurse who assisted her at an operation on her patient, Mrs. Byrne, to exercise due care in the performance of her duties. In such a case it naturally devolves upon the nurse in attendance to count the number of sponges in use, and to check the number when they are finished with. It was affirmed in this instance that the counting had been done both before and after the operation; but if so, it must have been done so perfunctorily as to render it valueless. Obviously a nurse falls short of trustworthiness who cannot be depended upon to be accurate in even the smallest details; and if the higher training of nurses is to render them neglectful in matters of this kind the effect will in the end be disastrous.

### THE STOCKPORT HOSPITAL SCANDAL.

There is one incident in connection with the storm which has been raging respecting one of the Stockport Borough hospitals that requires a word of notice. In the course of a discussion at a meeting of the Town Council, Dr. Hyde Marriott, a member, contended that it was the duty of the medical superintendent of the hospitals to look after the soiled linen which was left on the bed in an observation ward. Whatever may have been the motive for this assertion, it was absolutely inaccurate, and, coming from a medical man, extraordinary. It is, of course, one of the regular duties of the matron of a hospital to see that all the linen is washed at the proper time.

### THE PRECAUTIONARY REGULATIONS OF THE ASYLUMS BOARD.

With reference to a statement made by a correspondent that a trained nurse recently left one of the hospitals under the jurisdiction of the Metropolitan Asylums Board without any of her clothes being disinfected, we learn that in this instance an investigation is precluded by the fact that the hospital in question was closed some months ago. As we concluded, however, the precautionary regulations of the Board on this point are stringent. We are indebted to the courtesy of the clerk for a copy of the manuals of the rules which the nursing staff at the fever and small-pox hospitals are required to observe. They are directly forbidden to leave the hospital premises at all, except when employed on ambulance duty, without having first changed their uniform clothing and stockings. No nurse is allowed to sleep away without having first changed all her wearing apparel, and, except in case of exemption by the medical superintendent or matron, taken a bath; and all nurses, upon leaving the service of the Board, are required to satisfy the matron that their clothing has been cleaned and disinfected, and that they have taken a bath. We are informed that in December 1902, a special sub-committee of the hospital committee of the Board, having fully considered the question, reported to the effect that the replies received from the general hospital sub-committees in regard to the staff regulations respecting disinfection before leaving the premises satisfied them that the regulations are carried out satisfactorily and without undue inconvenience to the staff. The case mentioned by our correspondent was, of course, subsequent to the issue of this report, but we regret that it was not brought under our notice at the time it is alleged to have occurred. The precautionary regulations of the Board, admirable as they are in other respects, do not give instructions as to where the in and outdoor clothes of nurses shall be kept. According to statements by two of our correspondents, it appears to be the practice merely to exclude uniform from cupboards or drawers where the outdoor clothing is placed. This is not an adequate precaution, and we suggest that the resources of the Board impose upon them the duty of providing separate rooms, quite apart from bedrooms or wards, where all outdoor clothing could be kept.

### EXAMINATIONS BY MATRONS.

The appointment of the matron of Lambeth Union Infirmary to conduct the examination of the probationers at Bermondsey Union Infirmary in practical nursing, is noteworthy, and will doubtless be emulated by other Guardians who desire to promote the efficiency of training. Miss Byles is to receive a fee of five guineas for her services, and so long as a matron's outside work does not interfere with the discharge of her duties in the institution to which she is attached, there is no reason why she should not be allowed to render assistance elsewhere.

### A NURSE ACCUSED OF BURNING THE FEET OF A PATIENT.

An action was brought in the Pocklington County Court, Yorkshire, on Thursday last week, by a labourer and his wife against Mr. J. J. Bickersteth and Lady Margaret Bickersteth, as treasurer and secretary respectively of the Wilton Beacon Benefit Nursing Association in the East Riding. Damages were claimed on the ground of negligence on the part of a nurse towards the female plaintiff. Evidence of a voluminous character was given, but in the result the judge decided that no case of negligence by the nurse had been proved, and though that disposed of the question, he added that the Association being of the character of a friendly society, the action against it could not be sustained. It is remarkable that while the nurse was accused of burning the feet of the patient by the application of a hot-water bottle after the operation, no one, according to the newspaper report, asked where she was trained. Two doctors who were examined, however, expressed the opinion that a fully-trained nurse ought to have been employed; and while we observe that the surgeon who was in charge of the case said that he "did not require the services of a highly-trained nurse," we agree with the other medical men. In justice to the nurse employed, it should be stated that before she went to the case she did not know that there was to be an operation; but it is a sound principle that if a serious operation on a patient is considered requisite, a fully-trained nurse should be in attendance.

### THE CO-OPERATION OF THE CLERGY.

An interesting meeting has just been held at Sheffield with the view of specially enlisting the sympathy and support of the clergy for the Queen Victoria Nursing Association. The feature of the meeting was an able address by Alderman Franklin, who claimed that the work in which the nurses are engaged is akin to that of the clergy, and that therefore the co-operation of the latter should be warmly given. In most towns this co-operation is not withheld, but in Sheffield, it appears, it has not hitherto been so largely accorded as it might be. Alderman Franklin states that to cover the whole city about 40 Queen's nurses are required, instead of 21, the existing number, and we hope that the resolution, which was subsequently passed unanimously, binding the clergy present at the meeting to help financially, "either by collection or in some other way," will have the result of enabling the association to increase the staff at an early date.

### A WRONG TITLE.

At a meeting of the Pontardawe Guardians the applications of three candidates for the appointment of a nurse in the Workhouse were discussed. It being objected by Dr. John Jones that a salary of £25 a year offered was insufficient for a trained nurse, Alderman Jones observed "The title of nurse is wrong. It isn't nursing she has to do; she's more of a general servant than a nurse. When girls are put to scrubbing they soon look out for another place." The Alderman is perfectly accurate as to the title of the person whose services he wishes to obtain. The title of nurse is not convertible with that of general servant, and the Portardawe Guardians do not fulfil the requirements of the Local Government Board by advertising for a nurse and engaging an individual to act as a general servant.

### LEEDS NURSES AT PLAY.

A pleasant afternoon was spent by part of the nursing staff of the Leeds Union Infirmary on Tuesday last week at Adel, a charming village a few miles out of Leeds. About thirty were entertained by Mrs. Arthur Baines, of "The Heath." Two charabancs were sent to the Nurses' Home for them, and, as most of them were in their outdoor uniform, they were ready to time, the start being made at 3 p.m. Some were rather doleful at the outlook of the weather, which was dull and drizzling, but before long the rain considerately ceased, allowing the party to wander in the beautiful gardens. The profusion, as well as the rarity of the flowers, delighted town-accustomed eyes. Tea was served in the hall of the house, and subsequently, as the rain fell again, it was impossible to carry out the original plan of visiting the various places of interest adjacent. Accordingly, while some of the guests stayed in a roomy summer-house puzzling each other with conundrums, string tricks, etc., others inspected the old Norman church, and the organist kindly played a few selections. Returning to "The Heath," all joined in parlour games, and, after music and refreshments, the nurses said a grateful good-night to their hostess, and started home, each with a bunch of flowers and feeling quite indifferent to the incessant downpour.

### STRANGE PRESENTATION TO A NURSE AT STORNOWAY.

In the *Aberdeen Free Press* of May 11, there appeared a paragraph to the following effect:—"Millicent Gwendolen Mary Whawell, who arrived from London in February last to be district nurse at Ness, Lewis, left the island on April 28th, and was arrested in Wigan by the Lancashire police, and brought back to Stornoway. Yesterday, before Sheriff Squair, she was charged with having fraudulently induced Mrs. Murray, Habost, Ness, to give her board and lodgings, and with the theft of several small articles. She pleaded not guilty to several of the counts, but was convicted on evidence of having appropriated two tea-cloths, fraudently obtained from Mrs. McKay, wife of the lighthouse-keeper of Butt of Lewis. The Sheriff extended to her the benefit of the First Offender's Act, and bound her over to be of good behaviour for six months." In the issue of the same paper for May 20, it was announced that "Great interest has been evinced at Stornoway, since the fishing commenced, in the case of Nurse Whawell, who was, on May 19, acquitted of the charges brought against her. A number of gentlemen from the East Coast, to show their sympathy towards the lady under trying circumstances, met the same evening at the Caledonian Hotel and presented her with a purse of sovereigns as a mark of their sympathy. Mr. Thomson, fish salesman, in making the presentation, said he was sure from what they had heard of Nurse Whawell since she came to the island that her character and abilities were beyond dispute. As her solicitor said in court that day, the charges brought against her were of the most flimsy and trivial nature. He was sure that these sentiments were entirely endorsed by all the subscribers to the testimonial. They wished her every success in the profession in which she had embarked, and the purse of sovereigns which he had the pleasure of handing to her was only a small tribute of the respect in which she was held by the subscribers. Miss Whawell's solicitor returned thanks on her behalf."

### THE NURSES OF BRISTOL GENERAL HOSPITAL.

It is a feather in the cap of the Bristol General Hospital that Miss Mabel Fragbery, lately appointed sister to the Norwood Hospital for Consumption; Miss Stevens, matron's assistant to the Hampstead Hospital for Consumption; Miss E. Davies, sister at the Weston-super-Mare Hospital, were all trained at that institution. Miss Fragbery possesses the gold medal of her training school, and Miss Stevens the silver medal. They also both hold the L.O.S. certificate, and gained their midwifery training at the Bristol General Hospital.

### CHARGE OF INCENDIARISM.

At Marlow, on Tuesday, a nurse named Isabella Jane Livingstone was committed for trial on a charge of setting fire to the Isolation Hospital at Brooker, belonging to the High Wycombe Corporation, bail being allowed. She had been remanded for a week, and was brought into court struggling with the female warder. During the reading of the charge she shouted, "Put it out, put it out," and while evidence was given she wept bitterly. The motive for the alleged offence is that the nurse was annoyed that she was not appointed matron of the hospital.

### SHORT ITEMS.

In commemoration of Queen Victoria's birthday, Mrs. MacDonald Stuart of Dulness Castle, Glencoe, entertained a number of Queen's Nurses to lunch and tea, and her kindness was warmly appreciated.—The Stepney Guardians consider that £3,305 is too large a sum to spend on the provision of a nurses' home at Bromley Workhouse, and they have therefore asked the architect to submit some less elaborate plans.—The social gathering for nurses at Brighton, organised by the Dowager Countess of Tankerville, will take place on Friday, June 17, at the Royal Pavilion.

### The Nursing Outlook.

"From magnanimity, all fear above;
From nobler recompense, above applause,
Which owes to man's short outlook all its charm."

### THE NURSE-EVANGEL.

To be both a doer of good deeds and a bearer of good tidings! Such is the high aim of the missionary who is a trained nurse, and such an aim has been put very strongly before the "Nurses' Missionary Union" this week at their annual conference. The meeting was held at the Church Home of All Souls, Langham Place, on Monday, with Dr. Henry Soltau, of Burmah, in the chair in the afternoon, and with Mr. Percy Paton, of Westminster Hospital, in the chair in the evening. Two nurse-missionaries were amongst the speakers, and told of the difficulties of labour in far-off lands—and also of the delights, for the whole tone of the meetings was cheerful and full of the splendid optimism which marks the motto of the Union—"The Evangelisation of the World in this Generation."

The Nurses' Missionary Union was only formally inaugurated in 1903; and it claimed as its reason of formation that during the last 50 years the Church of Christ had shown two remarkable developments—first, the increase in medical missions, and secondly the wider employment of women in all departments of Christian work. Its members are nurses who hope to echo Miss Havergal's cry—

"Take my life and let it be
Consecrated, Lord to Thee,"

and its associates are those nurses who desire by prayer to forward the Evangelisation of the world.

The Union employs a travelling secretary to be at the service of nurses, in giving advice with regard to the organisation of branches of the Nurses' Missionary Union and the development of the Missionary spirit, in arranging joint meetings between hospitals, and making possible an interchange of experience and sympathy, in suggesting methods of study and preparation for Christian service, in making known the needs of Mission work at home and abroad, and information with regard to such service.

And there are many other societies whose work is in the same direction: our advertisement columns constantly bear evidence of the call for Bible-women, for Church Army nurses, for Mildmay nurses. The work of the nurses of the Zenana Mission has been frequently noticed in our pages; and we have, too, a vivid appreciation of the work done in the slums by the Salvation Lass, though her training is often very limited. Of course the professional aspect is to look down upon the nurse-evangel; the woman who says a prayer as well as makes a poultice, and this is not unnatural, because many of these nurses have been so immersed in their message that they have been content with but little information about nursing. That is a mistake. "Thorough" is the best motto for us all, whatever our religion, thoroughness in our work and force in our convictions. Then on their side the missionaries have been struck at times by the lack of sympathy and the littleness of thought of the trained nurse thrown amongst the big questions of the East:—"They just work their duty hours, and then they go gallivanting to balls or go off to picnics—they don't seem to care for the people or the country in the least," wrote a missionary lately. There are always two sides to a question, and it is very difficult for any one person to see both.

But exactly as we have advised the professional nurse in these pages to join some league or society, or in some way keep in touch with her comrades, her hospital, and the nursing questions of the day; so we think it is well for the nurse-evangel to have some society, for there is great force in numbers and it is not easy to stand alone. The interchange of ideas is always stimulating; the knowledge that others have trod the same difficult path is always inspiring; we can all help one another by sympathy if not by words. And it is quite possible that if the missionary nurse and the professional nurse came in contact they might learn from one another the value of training and the value of high aims. The routine life of hospital is apt to pall upon a nurse, apt to deaden her susceptibilities. In "Olive Lathom," a powerful novel lately published by Mrs. Voynich, this is shown in no unkind manner. After seven years in hospital the daughter returns home for a rest, and the eager father who has longed for this reunion tries to open his mind to her and tell his trials. The next morning, when he goes down to breakfast, he finds a little bottle of pepsin tabloids beside his plate. Such is Olive Lathom's expression of sympathy! Subsequently, under severe suffering, the crust is broken through, and the woman emerges once more. Olive finds the inspiration which sets her soul free in the Nihilist movement in Russia; probably any other great movement would have done equally well, anything to take her out of the rut into which she had fallen. And therefore it is that the enthusiasm of the nurse-missionary strikes us as very fine, and recalls to us one of Miss Lückes' favourite sayings, "Not failure, but low aim is crime." Here in conclusion are some lines, and the present writer would be glad if any reader can name the author:—

Lord, let me not be too content
With life in trifling service spent—
Make me aspire!
When days with petty cares are filled,
Let me with fleeting thoughts be thrilled
Of something higher!

Help me to long for mental grace,
To struggle with the commonplace
I daily find.
May little deeds not bring to fruit
A crop of little thoughts to suit
A shrivelled mind.

### The Modern Nursing of Consumption.

By Dr. Jane H. Walker, Physician to the New Hospital for Women, London, and Medical Superintendant of the East Anglian Sanatorium, Nayland, Suffolk.

### LECTURE VII.

### The Patient's Day.

*Food and Rest.*—Private patients are apt to rebel most against this part of the treatment. It will require all a nurse's powers to make a patient and his friends believe that three full meals a day can be both eaten and digested, although he may be taking little or no exercise.

Diet must, of course, depend upon the doctor's orders. An ordinary daily *régime* would be: Breakfast at 8.30, consisting of tea or coffee, milk, porridge and cream, and bacon and eggs or fish, or some such dish, with bread or toast, and about an ounce of butter.

After a rest of an hour the patient may be washed (we are now considering a case with high temperature and a rapid pulse); then he should lie quiet till 12, receiving perhaps a visitor, or being read to by the nurse. From 12 till 1 he should be absolutely quiet, not even talking. At 1 he should have dinner—soup, fish and potatoes, meat and vegetables, pudding and cream, and biscuits and butter.

During the afternoon, if the doctor allow it, and the temperature and pulse be not raised (in comparison with previous days) he may have a visitor, the nurse may read aloud, or he may play chess or draughts. At 6 again an hour's absolute quiet should be enjoined, and at 7 comes supper. This may consist of fish, poultry or game, or meat only, with vegetables, followed by pudding and custard, or cream with milk to drink.

At 9 the nurse should again wash the patient and make his bed for the night. If well enough he may be on the couch while this is being done. By 10 P.M. he should be settled in for the night.

There are ways and ways of taking meals to a patient. In the first place the tray should be big enough to contain all requirements, and the tray cloth should be absolutely clean. There should be no such thing at dinner as a cloth stained with the coffee of the previous meal, nor at breakfast traces of the gravy of the night before. This may seem self-evident, and it may appear unnecessary to draw attention to such trifling details, but the point is distinctly important, and I know from personal experience that it is one frequently overlooked. Next see that the patient is comfortably settled in bed, at an angle convenient, not to the nurse, but to the patient. Here I know that I am treading on delicate ground. For if a nurse knows anything she knows how to smooth the ruffled pillow, and cool the fevered brow, so to speak. Then see that the patient has everything that he wants, salt is not infrequently omitted, remember too that some people take sugar with porridge, and others take it with tea and coffee. In fact a nurse should put herself as far as possible in the patient's place; in other words she should be really sympathetic, without being soft and mawkish.

Many patients begin to cough as soon as they begin to eat, this often results in a feeling of intense nausea, if not in actual sickness. A good way to prevent this is to give a tumbler of very hot water to be sipped slowly, half an hour before each meal, in this water, if the doctor sanction it, a few grains of bicarbonate of soda and the same amount of common salt may be added. This hot alkaline drink will generally soothe the throat, and if the patient is going to have an attack of coughing he will get it over before the meal arrives.

The same attention to making the bed, and settling the patient for the morning, if he be able to be up, must be paid as in the case of patients in sanatoria.

Patients who are allowed to get up will of course take what exercise is prescribed by the doctor, the nurse making such alterations as she thinks required by the patient's condition. For instance, if he be feeling very tired, or the temperature have gone up to 100° F. the night before, or if it be not normal (or below) in the morning, she might keep him in bed for the morning, or not allow a walk, or might cut off visitors for the day. The same absolute rest before dinner and supper will be required, and the meals will be much the same as if he were entirely in bed. In many cases a doctor will order rest after, as well as before, meals, and the nurse must see that rest hours are *really* rest hours and not broken in upon by friends and visitors. A little blood in the sputum is an indication for rest; so is a quick pulse, sickness, headache and pain in the side, or extra cough or shortness of breath. The temperature and pulse should be taken four times a day, in the early morning, during each rest hour, and at night. She must remember in the case of patients who are up that there must be no sitting about in stuffy dining or drawing-rooms after dinner. A patient who lives in the open-air gets very sensitive to a stuffy atmosphere, and half an hour before bed in a hot sitting-room will generally mean coughing half the night afterwards.

When taking exercise patients should walk at "sanatorium pace," that is two miles an hour. Driving may be permitted if there be no wind; here again the doctor may say a patient may have a drive, but if the nurse find a gale blowing when the time comes she must veto it and explain to the doctor at his next visit why she did so.

The nurse herself should get some free time every day, besides the rest hours, and should have a good brisk walk or cycle ride daily. She must, if not blessed with one naturally, cultivate a good appetite, to set an example to the patient, and to keep herself physically fit. If the patient have to eat well, and to finish all he is given, there must be no toying with food on the nurse's part.

A daily and weekly diary should be kept for the doctor's benefit; an observant nurse, seing the patient daily, can give the medical attendant much help, and a nurse who is accurate and observant, and has had experience in chest cases can generally tell fairly well what rises of temperature, quickening of pulse, much cough or fatigue are due to, and know when it is necessary and when unnecessary to send for the doctor, and often can thus spare both patient and friends much anxiety and worry.

We have referred many times to the patient's friends. It is really often the friends and relations who militate most against recovery. One of the chief advantages of a sanatorium is that the patient is taken away from home surrounding, from the constant excitement of the "cheering" process so very generally thought necessary, and from the anxious, if sympathetic, looks and words of friends and companions. Phthisis patients suffer from a disease which produces not only local but constitutional effects. Not the least of these is shown in the temperature and pulse. The nervous system is unstable, and very little excitement will raise both temperature and pulse. One of the difficulties of treating patients in their own homes is that of inducing both patients and their friends to believe that it is rest of mind, as well as body, that is needed. The nurse will in a private house have to steer a difficult course between being a dragon and being too lax and allowing visitors when the patient is really unfit to receive them. Boredom is most wholesome for phthisis patients. The hectic flush, beloved by novelists, the quick pulse and night sweats, are all, we believe, largely due to over excitement of the nervous system, and unless the whole system can be kept quiet and at rest the patient is unlikely to make any real progress.

### The Editor as a Patient.

BY ONE OF THE NURSES.

THE carriage accident which befell the Editor of THE HOSPITAL on Whit Monday at Thrapston, in Northamptonshire, has brought prominently into notice the practical absence of all provision for handling such cases in the country properly and efficiently.

The accident occurred owing to the horse in the waggonette in which Sir Henry Burdett was seated taking fright at a motor car and bolting. In the result all the occupants of the carriage were thrown with tremendous force on to the rough uneven ground beneath the hedge. Our patient was the most severely hurt as he was sitting in the front of the carriage, which turned first on its side and then completely upside down. He was rolled over by it and very severely knocked about by portions of the carriage striking him. There were fractures of the neck of the left clavicle and scapula, a fracture of the head of the humerus which was impacted and involved the shaft of the bone, so that the whole of the bones entering into the left shoulder joint were involved. Some ribs were fractured also. The amount of extravasation throughout the body was very great, and produced, with the other lesions, the most excruciating pain. Indeed, when I arrived, I found that we could hardly touch the patient anywhere, and there appeared to be only one small place on the right side where he could rest with any degree of ease.

Our patient was full of useful resources, but, owing to the great difficulty caused by the absence of appliances and the unavoidable delay in getting them from London, it was practically eight days before he could get any natural sleep or any relief from his intense sufferings. Such a long delay was caused because the first patent bed which was sent proved to be an utter failure, and it was not until we obtained one of the new bedsteads in use at Fitzroy House and Guy's Hospital—made under the direction of Sir Cooper Perry—that our patient obtained a certain degree of comfort which ensured some relief from pain and some hours' sleep.

Fortunately there were no complications, only the continuous and acute suffering, the absence of sleep, and the presence of so large an amount of extravasation that its reabsorption produced evidence of toxic poisoning. These symptoms we have now been able to master, and the patient is slowly but steadily progressing towards recovery. The hæmatomata are greatly diminished in size though not at present in their powers to cause suffering, and the general condition is in every way satisfactory.

The Editor is very much impressed by the need in a country district of a complete set of appliances; first for moving the patient from the place of the accident to his home, which in the case of the Editor was a difficult problem, involving a wait on the road side of over an hour, and then again another hour in a motor car until some kind of carrying chair could be improvised to convey the patient from the car to the bed which had been hastily put up in a room on the ground floor; and secondly for the necessary fracture bed with pulley, etc., for giving the patient the rest he requires after the shock of a bad accident.

Our patient is greatly concerned by all this, and although we have done our best to keep him quiet and prevent him from using his brain, we are fairly confident that he has already evolved a plan for providing every country district with a complete set of appliances which will render it impossible for other victims of accidents to again experience the volume of suffering which has necessarily fallen to his lot, owing to the absence of anything of the kind at the present time in any country district.

### Plaistow Maternity Charity and District Nurses' Home.

THE eleventh annual meeting of the Plaistow Maternity and District Nurses' Home took place on Friday last in the garden of the Home at Howard's Road, Plaistow. There was a good attendance, including two large groups of the nurses belonging to the institution, and the Hon. Mrs. C. Egerton presided.

The Bishop of Colchester, chairman of the committee, in moving the adoption of the report, commended the claims of the institution to all who further the cause of nursing the poor, and pleaded for subscriptions for this charity on the ground, first, of its local work in the southern parts of West and East Ham; and, secondly, of its national work in providing nurses and nurse-midwives for the rural poor. He thought that perhaps only the clergy and doctors knew the real value of work done by such a charity.

The Mayor of West Ham, in seconding the resolution, said that public men could also bear witness to the excellence of the work done by the Institution. Having been born in the neighbourhood and lived there all his life, he had been able to watch the labour of the nurses, and he wished to give a special testimony to Sister Katherine, the lady superintendent, herself.

Miss Amy Hughes, who supported the resolution, remarked that the work of the Plaistow nurses would bear comparison with the work done by any other nursing school. Thoroughness, she said, and a very high standard characterised all that they undertook. Notwithstanding all the good public nursing lectures might do, they must take a back seat compared with the influence exercised by the nurses in the homes of the people. There was a large open door for good country women to be trained and live as nurses among the country women; and domestic nursing, with simple motherly talking, was what was needed. Miss Hughes also referred to the Midwives Act, and said that country associations, when sending nurses to be trained, should remember that more time must be allowed them in future.

The motion was carried.

Canon Buckley then moved a resolution conveying the hearty thanks of the meeting to the consultants and other honorary medical officers of the charity for their services; and also expressing its sympathy with the relatives of the late Dr. W. S. Playfair, and its sense of the benefit which the Institution derived from his skill and judgment during his long association with its work.

Dr. Garrett-Anderson, in seconding the motion, said that the work of the institution was most satisfactory. She considered that the tremendous field of work offered training advantages to nurses which could not be over-estimated.

The second resolution having been carried, Sir James Crichton-Browne spoke strongly in favour of the charity. He said that nurses were evangelists, and the indirect results of their work must be great. He went on to refer to infant mortality and pointed out that nurses can help the lives of the children by giving instructions—especially with regard to the feeding of babies—to the mothers.

Father Chappel, of St. Philip's Mission, said that he saw much of the nurses' practical work in his district and he simply did not know how they could get on without it.

At the close of the meeting the home and small hospital were open for inspection. Tea was served in the garden, and a prettily-decorated booth made a concert platform, whence proceeded welcome strains of vocal and instrumental music.

### Royal British Nurses' Association: Resignation of Treasurer.

Considerable disappointment was occasioned to members of the Royal British Nurses' Association on Monday afternoon when it was announced by Mrs. Latter that Princess Christian could not be present, owing to an important engagement at Buckingham Palace.

The chair was taken by Dr. Bezley Thorne, who was supported by Mr. John Langton, hon. treasurer; Dr. Comyns Berkeley, hon. sec.; Mrs. Latter, who has been appointed nurse hon. sec. during the temporary absence of Mrs. Coster in Canada, and Miss Hobbs, secretary.

The hon. treasurer's report, read by Mr. Langton, showed that the accounts had been closed at the end of the year, instead of, as previously, in March, so that the figures stood for the nine months ending December 31st, 1903. This, the Treasurer explained, had been done in order to bring the accounts into unison with business practice. The expenditure amounted to £483, and there was a deficit of £52 1s. 8d. Some expense had been saved in salaries, since the duties of registrar were being temporarily undertaken by Miss Hobbs and the clerk, Miss Lees. Donations amounted to £223 10s., without which the financial position would have been more unfortunate than it was. The Settlement Fund now possessed securities to the amount of £2,353 15s. 2d., in addition to £360 16s. 8d. at the bank. This represented an increase of £222 17s. 8d. over the figures for last year. A house had been secured at Clapton Square, Hackney, and the freehold bought and paid for, at a cost of £1,000. The few alterations necessary would cost not more than £150, and he hoped it would be occupied within a month. The total grants made by the Helena Benevolent Fund amounted to £52 9s. 6d., including one pension of £20. The total outstanding indebtedness of the Association, as shown in the balance-sheet, was £211 1s. 0d. Mr. Langton said that his chief anxiety was that some means should be devised to bring the income and expenditure into more direct harmony with each other, so that the finances might be placed on a sounder and more satisfactory basis. He moved the adoption of the financial statement.

Dr. Henry Woods seconded the motion, and in doing so dwelt on the devoted labours of the hon. treasurer; during his ten years of office, he had put into the work not only time and an immense amount of toil, but a large sum of money, and he urged the nurses, in view of the fact that so many people were trying to help them to improve their status, to do something to help themselves. If they did this, not only would the Association be in a much better financial position, but their increased efforts would show their deep sense of their obligation to the hon. treasurer.

The Chairman, having endorsed the remarks of the last speaker, put the motion, which was carried unanimously.

Dr. Comyns Berkeley read the report of the executive committee, dealing largely with the progress of the Bill for promoting State Registration of Nurses. It had been decided, on grounds of economy, to reprint the roll of members once every three years, and to issue, in the other two, addenda containing the names of new members, with any deaths or resignations that might have occurred, and to delete addresses. During the year ending May 31st, 1904, 117 nurses had applied for registration, of whom 108 were accepted. One hundred and eighteen new members were elected during the same period, 53 had withdrawn, and there had been 21 deaths.

Dr. Henry Hawkins, in seconding, said it was evident that the Association had been very hard at work, and keeping well to the front in all matters relating to the interest of nurses. He believed the majority were in favour of some form of registration. The report was adopted.

A resolution was brought forward by Miss Beatty, calling upon those honorary officers who had signed the manifesto against State registration to resign their membership of the Association. This was seconded by Dr. Bedford Fenwick, who, in the course of his remarks, informed the meeting that within an hour of that time the appointment of a Select Committee of the House of Commons, to inquire into the nursing question, had been consented to by the Prime Minister.

Mrs. Dacre Craven and a Nurse spoke in emphatic terms of the valuable services of the honorary officers referred to, and said that they could not be spared by the Association.

The Chairman observed that while the feeling of the Association was overwhelmingly in favour of registration, tests in matters of opinion had never been imposed on members; everyone had a right to think for himself and herself.

The resolution was lost by a large majority—72 voting against and 2 for the motion.

The announcement that he intended to resign the office of hon. treasurer was then made by Mr. Langton and received by the nurses with evident surprise and regret. Mr. Langton said he felt the time had come when a younger man should take the office he had so long held. He did not deny that he had put an amount of work as well as money—£1,100—into the Association, and although he would say nothing as to the financial aspect, he felt unable to continue the work. He wished the Association every success in the future.

The Chairman and others having expressed the sense of profound regret as well as gratitude felt by the members, the meeting separated with votes of thanks to the chairman for presiding and to the authorities of the Imperial Institute for the use of the room.

### Dinner to American Nurses.

BY A CORRESPONDENT.

The American nurses, who are in London this week *en route* for the International Congress of Women at Berlin, were entertained at dinner at the Criterion on Monday night, the welcome being organised by a number of English nurses glad to seize such an opportunity of reciprocating the cordial greeting many had themselves received from their fellow-workers in the United States on the occasion of the Chicago Congress. It was unfortunate that instead of the 40 expected guests 10 only arrived in time to be fêted, but the hostesses mustered in force and the evening proved quite a success, about eighty sitting down to dinner.

Miss Isla Stewart presided, and amongst others present to greet Miss Mary Thornton and her party were Miss Peter, Miss Sidney Browne, Miss Amy Hughes, Miss Mollett, Mrs. Bedford Fenwick, Lady Helen Munro Ferguson, Miss Debenham, and Miss Hobbs. The London Concert Trio made pleasant music, and the usual speeches were of more than usual interest. Apart from the pleasant things to be said on such an occasion at any time, there is a specially friendly feeling between Anglo-Saxon peoples just now which found cordial expression in this assembly of nurses. Miss Thornton, who is secretary of the National Associated Alumnæ of Nurses of the United States, made an amusing reply to the speeches of welcome, assuring her hearers that though she and her party of delegates had been but two days in England, they had seen something already of Liverpool, Chester, Warwick, Stratford-on-Avon, Oxford, and London. They were all most heartily appreciative of the kindly hospitality with which they had been greeted.

### Colonial Nursing Association.

### EARL GREY ON THE FINANCIAL POSITION.

THE annual meeting of the Colonial Nursing Association was held on Wednesday afternoon at Sunderland House, and among the company present was Princess Henry of Battenberg, patroness.

Earl Grey, Chairman of the Executive Committee, who presided, was supported on the platform by the Duke of Marlborough and the Earl of Westmeath, and among the ladies present were the Duchess of Marlborough, Lady Balfour of Burleigh, Lady Norman, Lady Ommanney, Mrs. Chamberlain, Mrs. Frances Piggott, Miss Rosalind Paget, Mrs. Latter, Miss Dalrymple-Hay, and others.

The Chairman said the Association was now fulfilling the *rôle* and had assumed the dignity almost of an institution of the State. Of the 109 nurses now at work, 81 had been selected at the request of his Majesty's Government for work in the Crown Colonies, and the mere fact that the Association had now a large staff working with the full co-operation and approval of the Colonial Office constituted, in his opinion, a claim on every public-spirited individual, a claim to which he hoped there would be a quick response. The demand from various parts of Greater Britain for qualified nurses was continually increasing, while, on the other hand, the number of nurses with full qualifications anxious to place their services at the disposal of the Empire was also continually increasing. To enable the Association to fulfil the high objects it had in view, an appeal to public sympathy and support was absolutely necessary. There was nothing of a pauperising nature about the Association; while it worked in co-operation with the British communities across the seas, it required a large floating capital to enable advances to be made for outfit and for the payment of passages as well as to meet the increasing office expenditure rendered necessary by the growing dimensions of the administrative work, and also to defray the cost of the medical examination of candidates. The Association was most anxious to secure additional funds also, to enable a pension fund to be established, upon which those who fell in the battle might draw. At present each colony had its own pension rules, but when a nurse was transferred from one part of the world to another she lost her claim on the funds. It was desired to establish an Imperial Fund, which would enable the services rendered in one colony to count for a pension even if the nurse were transferred to another. This was a form of assistance everyone would wish to render to the gallant and self-sacrificing ladies who went out at the risk of health and life in the service of the Empire. The need for such a fund was self-evident. He ventured to prophesy for the Association an ever-expanding sphere of usefulness in helping to build up and consolidate the British Empire abroad.

The Duke of Marlborough moved the adoption of the report. As Under-Secretary of State for the Colonies, he spoke of the evidence continually received there of the value of the Association's work, and the admirable nature of the nurses' services. He hoped adequate support would be given by all who had the true interests of the Empire at heart.

Lord Westmeath, in seconding, said he would like to see the Association a centre of Imperial activity.

The motion was carried unanimously.

Lady Lugard, Sir George Carter, K.C.M.G., and Sir Colin Scott-Moncrieff, K.C.S.I., also spoke, from personal experience, of the excellent work of the nurses in the Crown Colonies and Protectorates, and urged the need of increased financial support.

The election of new members of the Council, and of officers approved by the executive committee, was proposed by Sir Charles Bruce, G.C.M.G., and seconded by Sir Matthew Nathan, K.C.M.G.

Earl Grey proposed a vote of thanks to Princess Henry of Battenberg for her presence and continued interest in the Association; and votes of thanks to the Duke and Duchess of Marlborough, and to the Chairman, closed the proceedings, to which there was an added interest in the presence at the door, with plates for contributions, of the two little sons of the Duke and Duchess, Lord Blandford and his brother.

### Everybody's Opinion.

### STATE REGISTRATION.

"A MATRON" writes: I should like to ask what State registration is going to do for the large section of nurses who, having had their three years' training and obtained their certificates, close their books, lose their enthusiasm, and gradually deteriorate; who know less at the end of their six years than they did at the end of their three? It has surprised me not a little that so many nurses have an idea that there is nothing more to learn when once they have obtained their certificates. To ask them to attend a lecture when once they are certificated is looked upon as a reflection upon their knowledge. They rarely ever make use of the hospital library, or, if engaged in private nursing, seldom buy a medical book, and after a time seem averse to adopting new methods, freely criticising the medical staff and making comparisons with the treatment adopted in their time. Many who have been responsible for the training of nurses cannot fail to have observed the difference there is in those who enter the large training schools. There are those who get through somehow, and there are those whose enthusiasm no amount of hard work can quench and who know more at the end of one year than the others will know at the end of three. They are interested and the others are not. They are thorough on all points, proving by their thoroughness in little things—a tidy kitchen, a neat linen or medicine cupboard, spotless lavatories and utensils, attention to the uninteresting patients—that they are to be trusted in the more important duties connected with their work. This type of nurse will be always learning, and her knowledge will always be bearing fruit. As a rule these nurses are quiet, reserved, unostentatious, they give themselves time to think, to lay up the knowledge they acquire, so that when the time comes to make use of it, they hardly remember the time they did not know it, consequently, having acquired the talent of quietly thinking, they never seem to be taken unawares, and they inspire confidence in the patient and implicit trust in the doctor. Will registration test any of these qualities? What is it to do for this type of nurse? What is it to do for the other class?—those whom no amount of training will make thorough, whom no one can persuade to consider the patient or the varying moods of the patient, who carry out the doctor's orders certainly, just as a machine would, but neither intelligently nor considerately, who themselves confess that a few years of private nursing makes them slack, keeps them in a groove, and who say there is nothing more to learn, and that even if they had the opportunity of returning to hospital work they would not, because they have forgotten nearly all they ever learnt. Can registration guarantee that, say three years after a nurse has been entered on the register, the medical profession or the public can with confidence say they are engaging either a well-trained nurse or a satisfactory nurse, any more than the registration of medical men can vouch for the reliability of a medical man. Certainly it guarantees that he has passed certain examinations, but it does not withdraw his name because he passed the one least difficult and took the lowest degree obtainable; because he belongs to no learned medical society whereby he may continue to increase his knowledge; because he never attends a post-graduate course, nor makes use of any hospital, nor procures the latest edition of standard works, and who gradually loses the knowledge he once possessed, and drifts into something less satisfactory than the old unqualified assistant who passed no examination and whose name was found on no medical register. Many nurses have admitted that they learnt more after they were certificated than before, and if this is so may there not be the class who allow themselves to forget what they learnt, and gradually to deteriorate into a careless and indifferent class of nurse, and yet remain on the register? One of the strongest arguments in favour of State registration is that those private nursing institutions which are private enterprises and which are unscrupulous enough to employ untrained or semi-trained women who have been dismissed as unsuitable from hospital simply because they get them for a very small salary, would no longer be able to make their institutions profitable because the three-year nurse, whether satisfactory or unsatisfactory, will demand to be paid a proper salary. In this way the untrained or semi-trained will become eliminated from the nursing ranks. But it does not follow that the unsatisfactory nurse will be eliminated. For nearly four years it was my privilege to be connected with a large provincial hospital which had a large private nursing staff attached to it, and as one of the assistants to the matron it fell to my lot to send out the nurses to their cases. These nurses worked for many eminent surgeons and physicians, and although many of them were sent out at the end of their second year's training, there was rarely ever any complaint made that they did not know their work. Complaints were not unfrequent, but in almost every instance these complaints related to the manners of the nurse, her untidy ways, or to the amount of attention she exacted from the servants, her want of considerateness to the relations. I remember perfectly some of the complaints—"We sent for a nurse, but have no use for a fashion-plate." "So far as nursing the patient was concerned, Nurse gave every satisfaction, but her personal habits were very objectionable, such as emptying tea-leaves down the W.C. and into bedroom utensils." "We are grateful to nurse for all she did for our invalid—and would willingly have kept her longer, but nurse objected to do those little duties that would have been so helpful, and preferred to spend the greater part of her time doing her own fancy work." The patient I knew was the mother of a large family of little children; would it not have shown a kindly considerateness on the nurse's part if she had offered to darn some of the little socks? after all, the patient paid for the nurse's services and time, she did not pay for the nurse to do her own fancy work when on duty. I could enumerate many more similar instances. I have been told that in Cape Colony, South Africa, where there is State registration, the standard of nursing has not been materially raised thereby. That nurses may cull a little knowledge from several different hospitals, and having totalled the time spent in hospital to two years may present themselves to the Medical Council for examination, and in the event of their satisfying the examiners are entered on the register and enter the ranks of the certificated nurse and compete with the fully trained. I have not had the opportunity of verifying this statement. To sum up briefly: A nurse having had three years' training and having obtained her certificate, with pleasant professional manners, with kindly considerateness, with neat exactness in dress, with loyalty to the medical profession, with a sense of the dignity of her office, will succeed wherever she may be placed. Without these qualifications it is somewhat doubtful if she will be a success even with State registration.

### Where to Go.

DOWDESWELL GALLERIES, 160 NEW BOND STREET.—An interesting exhibition is now open at these galleries of silver-work, enamels, and bronzes by Alexander Fisher.

GRAND BAZAAR, Prince's Skating Club, Knightsbridge, at 3 P.M., Thursday, June 9, Friday, June 10, in aid of the Hospital of St. John and Elizabeth, St. John's Wood.

### TRAVEL NOTES AND QUERIES.

By our Travel Correspondent.

SNOW MOUNTAINS (Natt).—Second-class return to Zermatt *via* Dieppe and Lausanne is £6 9s. 2d. If you decide to go by the Rhine you must inquire of Messrs. Cook the cost of that route. Why not stop at Lausanne going out for your friend to see that part of Switzerland, and at Berne or Neuchâtel on your return, which would not run up expenses. Hotel at Lausanne, Hôtel Faucon or Pension Campart, which has lower terms. At Zermatt, Hôtel d'Angleterre or Hôtel Gornergrat, from 6 fcs. At Berne, go to Hôtel Schmieden or Hôtel Pension Ruof. At Neuchâtel, Pension Borel, also called the Villa Surville, where terms are cheap. With care you can manage quite well on the money you have and can afford to make several excursions.

### Appointments.

CHRISTCHURCH WORKHOUSE INFIRMARY, HANTS.—Miss Mary Ford has been appointed charge nurse. She was trained at the Union Hospital, Stapleton, Bristol, and has since been sister at Aston Union Infirmary, Birmingham. She holds the L.O.S. certificate.

DUMFRIES AND GALLOWAY ROYAL INFIRMARY.—Miss Eleanor Steenson has been appointed night sister, and Miss Lizzie Strath surgical sister. Miss Steenson was trained at the Dumfries and Galloway Royal Infirmary, where she has since been night sister. Miss Strath was trained at the Royal Hospital for Sick Children, Aberdeen, and has since been nurse at the Dumfries and Galloway Royal Infirmary.

FRIMLEY URBAN DISTRICT ISOLATION HOSPITAL.—Miss Annie Edmonds has been appointed nurse-matron. She was trained at the Fountain Fever Hospital, Lower Tooting, London, and has since been nurse at the City Hospital, Little Bromwich, Birmingham.

GORDON HOSPITAL, LONDON.—Miss Edith Myers has been appointed matron. She was trained at St. Thomas's Hospital, London, and has since been sister at St. Monica's Hospital for Children, Brondesbury Park, and matron of the Queen's Jubilee Hospital, Earl's Court, S.W.

HEBBURN ISOLATION HOSPITAL.—Miss E. J. Smith has been appointed matron. She was trained at Monsall Hospital, Manchester, and Fulham Infirmary, Hammersmith, London, and has since done private nursing.

ISOLATION HOSPITAL, EAST MALLING.—Miss M. A. Hayles has been appointed staff nurse. She was trained at Milton Hospital, Portsmouth, and has since been charge nurse at Exeter City Fever Hospital.

KEIGHLEY UNION INFIRMARY.—Miss M. A. Fletcher has been appointed charge nurse. She was trained at Rochdale Union Infirmary.

MAIDSTONE SANATORIUM.—Miss Rosa Cooper has been appointed nurse matron. She was trained at the Borough Hospital, Wolverhampton, and the District Hospital, West Bromwich. She has since been assistant matron at the Sanatorium for Infectious Diseases, Huddersfield.

MORTLAKE ISOLATION HOSPITAL, BARNES.—Miss Margaret Baxter Clark has been appointed matron. She was trained at the Royal Infirmary, Aberdeen, and has since been head nurse at the East Poorhouse Hospital, Aberdeen, superintendent of nurses at the Infirmary, West Ham, and assistant matron at the Plaistow Fever Hospital.

THOMPSON'S MEMORIAL HOME, LISBURN, COUNTY ANTRIM.—Miss M. C. Tarrant has been appointed night sister. She was trained at the North Staffordshire Infirmary and Eye Hospital, Stoke-on-Trent. She has since been charge nurse at the South-Western Fever Hospital, London; private nurse at Hammersmith and Eastbourne; and head nurse at the Borough Hospital, Kingsthorpe, Northampton.

WEST HAM INFIRMARY.—Miss Daisy Crawford has been appointed sister. She was trained at St. Olave's Infirmary, London.

WESTON-SUPER-MARE GENERAL HOSPITAL.—Miss Edith Helena Davies has been appointed sister. She was trained at Bristol General Hospital, where she has since been staff nurse.

WINCANTON UNION INFIRMARY.—Miss Emma West has been appointed head nurse. She was trained at Stapleton Workhouse Infirmary, where she has since been assistant nurse. She holds the L.O.S. certificate.

"WOODLANDS" CONVALESCENT HOME, RAWDON, NEAR LEEDS.—Miss Frances Studdert Kennedy has been appointed nurse. She was trained at the Royal Hants County Hospital, Winchester.

### Echoes from the Outside World.

### Visitors to the King and Queen.

On Monday evening Prince Johann of Glucksburg, brother of the King of Denmark and uncle of Queen Alexandra, and the Duchess of Saxe-Coburg, widow of King Edward's late brother, journeyed to London from the Continent on a visit to the King and Queen at Buckingham Palace. The train containing the distinguished travellers reached Charing Cross between five and six, and the Prince and Princess of Wales, the Duke and Duchess of Connaught, and Princess Henry of Battenberg were at the station to meet them. The visit being a private one, there was no military escort. A considerable crowd assembled at Buckingham Palace to witness the arrival of the guests of their Majesties.

On Wednesday afternoon the Archduke Frederick of Austria arrived at Buckingham Palace. He has come to England in order to present to King Edward the insignia of a Field-Marshal of the Austro-Hungarian Army.

### Princess Mary of Hanover.

The death took place on Saturday morning of Princess Mary of Hanover, second sister of the Duke of Cumberland. The event was very sudden. The wedding guests, invited to the castle at Gmunden to be present at the wedding of Princess Alexandra to the Grand Duke Francis of Mecklenburg-Schwerin on Tuesday, were assembled at a reception when news came that the Princess Mary, who successfully underwent an operation for appendicitis the week before last, had lost consciousness. The party was at once broken up, and in a few hours her Royal Highness passed away still unconscious. Her life was devoted to attendance on her mother, the ex-Queen of Hanover, and her decease at the age of 54 is deeply regretted. The marriage, at the special wish of the ex-Queen, was not postponed, but took place as arranged in the presence of many foreign Royal and princely personages. The bridal pair left in the evening for Salzburg, but return on Saturday for the funeral of Princess Mary of Hamburgh.

### The War in the Far East.

An engagement between the Russians and Japanese cavalry at Li-chia-tun is reported. After two hours fighting the Russians retreated. A message was received at the Japanese Legation on Sunday night to the effect that Admiral Togo had heard by wireless telegraphy from the captain of one of his fleet cruising off Port Arthur that four masts with wireless telegraphic instruments on one, were seen on the top of Liao-ti-shan. On Saturday great explosions were heard and dense smoke was repeatedly observed to rise in the direction of Port Arthur. General Kuropatkin has reported to the Tsar a fight at a point 18 miles west of Feng-hwang-chenn, in which he claims that the Cossacks gained an advantage. Four thousand mounted troops from the Caucasus are on their way to the Far East. The Tokio correspondent of the *Times* says it is believed in Japan that the Russian squadron at Port Arthur intends to emerge at the crucial moment and fight for its life.

### The New Governor of Fiji.

The King has approved the appointment of Mr. Everard Ferdinand im Thurn, C.B., C.M.G., as Governor of Fiji, and his Majesty's High Commissioner for the Western Pacific, in succession to Sir Henry Moore Jackson, Governor Designate of Trinidad. Mr. im Thurn, who was educated at Marlborough and Oxford, became Curator of the British Guiana Museum in 1877, and held that post until 1882. He was subsequently stipendiary magistrate at Pomeroon for several years, and Government Inspector. From 1897 to 1899 he was employed on the Venezuelan Boundary Commission. Having next had two years' experience in the Colonial Office at home, he went to Ceylon, where, since 1901, he has been Lieutenant-Governor and Colonial Secretary. He is a great explorer, a most successful mountain climber, and has ascended Roraima, a feat which had not previously been accomplished. Mr. im Thurn's published works include "Among the Indians of Guiana," "A Tramp with Redskins," and "Botany of the Roraima Expedition."

### The Artists' Benevolent Fund.

An interesting feature in connection with the 94th anniversary festival dinner of the Artists' Benevolent Fund last Friday was that a lady presided. The lady was the Countess of Wemyss, and she ably discharged the duty of proposing the loyal toasts. After her husband had given the toast of the evening, Mr. Brock, R.A., proposed the health of Lady Wemyss, in complimentary language, and there followed later a delightful speech by Mr. Edmund Gosse, who replied to the toast of "Literature," Mr. Van Prinsep responding for "Art." The toast of "The Ladies" was responded to by the Duchess of Somerset in appropriate words, and ladies formed a considerable proportion of the company.

### Summer Fashions.

Now that spring jackets can be dispensed with, it will be seen that nearly every dress for summer wear is not complete without its accompanying little pelerine. These dainty small capes are made in any and every sort of fabric, but they are usually of the same material and in the same style as the garment itself, most of them ending in a point in the front. The white and cream muslin and lace gowns which will be so much in evidence this season, if the sun pays us a long visit, are nearly all provided with a fichu or pelerine trimmed with lace or narrow ribbon and ornamented with tucks or lace *motifs*. The greatest latitude is given in the matter of trimming for skirts. Flat bands to simulate deep tucks, a succession of small lace-edged flounces, or a deep frill as high as the knees—all these fashions are much in vogue. It is almost impossible to make sleeves too fanciful, and though some blouses are still without neck bands of any sort, many have lace round the throat, wired to stand almost to the ears, so that short fat necks and long thin ones should this year look equally well.

### Women Dramatists.

Two new productions at two of the London theatres within the last week have been from the pens of women. The first was "The Edge of the Storm" at the Duke of York's by Miss Margaret Young. The play is an ambitious one, with a prologue and three acts, the former taking place in Hungary, the latter in India at the time of the Indian Mutiny. By far the best portions of the play are those between the hero, Jim Poulett, a brave young Englishman, and Seta, daughter of Istvan, a Magyar patriot, who discovers after her marriage that her husband was unknowingly the murderer of her father. These scenes are played with fine feeling by Mr. Forbes Robertson, and Miss Gertrude Elliott, though falling somewhat short of the heights of tragedy, commands sympathy by her graceful and girlish performance. The mid-Victorian fashions are very effective. At the Camden Theatre on Monday evening "Warp and Woof," by the Hon. Mrs. Alfred Lyttelton, was produced before a fashionable audience, who had come from the West End to Camden Town in order to see what the dramatised version of "The Song of the Shirt" by the wife of the Colonial Secretary was like. The play is very interesting, and written in an earnest spirit, the second act being marked by considerable power. Mrs. Patrick Campbell undertook the part of the heroine.

### Notes and Queries.

**FOR REGULATIONS SEE PAGE 143.**

*Hospital Training.*

(88) Can you tell me if there would be any hope of my being accepted as a probationer? I have good testimonials, and a great desire to learn nursing, but a few months ago I served behind the bar.—*Anxious Nell.*

This fact need not disqualify you, provided that you are suitable in other particulars.

Is the Truro Infirmary considered a good training school for nurses, and is the certificate a good one?—*Hope.*

The Royal Cornwall Infirmary, Truro, is one of the recognised minor schools.

Is there any place where I could get free training in a children's hospital for a short time?—*E. A.*

Consult "The Nursing Profession: How and Where to Train."

Will you kindly tell me which hospitals give certificates of the most value to the nurses trained in them? Should I receive a thorough training in a local hospital?—*J. A.*

See "The Nursing Profession: How and Where to Train" for full particulars. The training in some of your local training schools is first-rate.

*Openings.*

(89) I shall be very glad if you will kindly tell me if there is an opening for an English nurse in Madeira or the Canary Islands. —*D. H.*

I am anxious to find a city where I might open a home for private nurses with some probability of success. Can you advise me? Is there a directory of such homes and institutions?—*H. K.*

See an article on "Private Nursing in Madeira" in our issue of March 5th last. The hotels in the Canary Isles usually employ as many nurses as are needed there. We never recommend openings. You can only find out what you want to know by inquiries on the spot.

*Male Nurse.*

(90) Will you tell me where a male nurse could be trained in medical and surgical work?—*M. L.*

1. I am not strong enough physically for work in an asylum, though possessing a good constitution; would you please inform me where I could apply for training as a male nurse or dresser? 2. What good text-book on male nursing can you recommend?—*P. R.*

1. The National Hospital for the Paralysed and Epileptic, Queen's Square, Bloomsbury, W.C., is the only civil hospital which trains male nurses. The Royal Army Medical Corps (18 Victoria Street, Westminster, S.W.) trains male nurses at the Government hospitals. 2. Any handbook for nurses would answer your purpose.

*Training in X-ray Work, etc.*

(91) Will you kindly tell me of an institute or hospital where I could become qualified in all branches of *x*-ray work and the administration of electric baths, etc.?—*J. C. L. M.*

You would probably gain most experience in one of the large private establishments devoted to this class of treatment. Messrs. Tallerman, 50 Welbeck Street, Cavendish Square, W.; or the Dowsing Radiant Heat Company, 24 Budge Row, Cannon Street, would possibly inform you of the more important institutions nearest to you.

*Convalescent Homes.*

(92) *Mrs. G.* will be glad to know the names of homes for convalescent children, either free or for a small payment.

See lists of Convalescent Homes in "Burdett's Hospitals and Charities."

**Handbooks for Nurses.**

"Nursing Profession: How and Where to Train." 2s. net.; 2s. 4d. post free.

"Nurses' Dictionary of Medical Terms and Nursing Treatment. By Honnor Morten. 2s. post free.

"A Handbook for Nurses." By J. K. Watson, M.D. 5s. net; 5s. 4d. post free.

"Practical Guide to Surgical Bandaging and Dressings." By Wm. Johnson Smith, F.R.C.S. 2s. post free.

"The Examination of Urine." By J. K. Watson, M.D. 1s. net; 1s. 1d. post free.

"Case-book for Private Nursing." 3d. net; 4½d. post free per copy; or per doz. 3s., post free.

The above works are obtainable of all booksellers, or direct from The Scientific Press, Limited, 28 & 29 Southampton Street, Strand, London, W.C.

### For Reading to the Sick.

"BARNABAS THE SON OF CONSOLATION, A LEVITE."

Comfort's true sons! amid the thoughts of down
That strew your pillow of repose,
Sure 'tis one joy to muse how ye unknown
By sweet remembrance soothe our woes,
And how the spark ye lit of heavenly cheer
Lives in our embers here,
Where'er the Cross is borne with smiles,
Or lightened secretly by Love's endearing wiles:

Where'er one Levite in the temple keeps
The watch-fire of his midnight prayer,
Or issuing thence, the eyes of mourners steeps
In heavenly balm, fresh gathered there;
Thus saints, that seem to die in earth's rude strife,
Only win double life:
They have but left our weary ways
To live in memory here, in heaven by love and praise.

*Keble.*

---

Sympathy, if real, must necessarily be allied with benevolence. We may not, indeed, be called upon to take up our abode amongst the poor, but we must hear of and inquire about them. We must seek out grief when it is hidden, as well as rouse ourselves to comprehend it when it is brought before us. We must practise sympathy in our daily life, not only by listening to small grievances and trying to place ourselves in the position which has made them grievances, but also by removing them so far as lies in our power. More than all, we must remember, what we constantly forget, and only half believe, that when we pray to God for the relief of suffering, we are in fact doing our best to mitigate it.—*E. M. Sewell.*

---

Our blessed Lord's first thought in the midst of torture was for others. So He teaches us unselfishness in cross-bearing. His perfect love pleads for His enemies their ignorance of what they are really doing. So He teaches us not to judge others hardly, not to judge them at all. He knows all that is in man's heart—we can only judge by appearances; how faulty, then, to say the least of it, must our judgment often be!—*R. W. Randall.*

---

There are some sorrows which we cannot share with others, and which must be borne by ourselves alone; yet we know that there is unspeakable comfort in having near us, even then, those who love us, and who, if they can do no more, can at least watch with us.—*D. Macleod.*

---

Sorrow is sent to teach us sympathy. We should try to comfort others "which are in any trouble, by the comfort wherewith we ourselves are comforted of God." What a talent for years to come is a sanctified sorrow! But if we will not use it, and persist in hiding it in the earth, we distrust God and we dishonour Him.—*A. W. Thorold.*

---

True love's the gift which God has given
To man alone beneath the Heaven.

. . . . . . .

It is the secret sympathy,
The silver link, the silken tie,
Which heart to heart and mind to mind
In body and in soul can bind.

*Sir Walter Scott.*

